# Harnessing the CRISPR/Cas9 system to disrupt latent HIV-1 provirus

**DOI:** 10.1038/srep02510

**Published:** 2013-08-26

**Authors:** Hirotaka Ebina, Naoko Misawa, Yuka Kanemura, Yoshio Koyanagi

**Affiliations:** 1 Laboratory of Viral Pathogenesis, Institute for Virus Research, Kyoto University, 53 Shogoin-kawahara-cho, Sakyo-ku, Kyoto 606-8507, Japan

## Abstract

Even though highly active anti-retroviral therapy is able to keep HIV-1 replication under control, the virus can lie in a dormant state within the host genome, known as a latent reservoir, and poses a threat to re-emerge at any time. However, novel technologies aimed at disrupting HIV-1 provirus may be capable of eradicating viral genomes from infected individuals. In this study, we showed the potential of the CRISPR/Cas9 system to edit the HIV-1 genome and block its expression. When LTR-targeting CRISPR/Cas9 components were transfected into HIV-1 LTR expression-dormant and -inducible T cells, a significant loss of LTR-driven expression was observed after stimulation. Sequence analysis confirmed that this CRISPR/Cas9 system efficiently cleaved and mutated LTR target sites. More importantly, this system was also able to remove internal viral genes from the host cell chromosome. Our results suggest that the CRISPR/Cas9 system may be a useful tool for curing HIV-1 infection.

Integration of reverse transcribed viral DNA into the host cell genome is an essential step during the HIV-1 life cycle^1^. The integrated retroviral DNA is termed a provirus, which serves as the fundamental source of viral protein production. HIV-1 gene expression is regulated by LTR promoter and enhancer activities, where cellular transcription factors such as NF-κB, SP-1 and TBP bind to promote RNA polymerase II processivity. Subsequently, Tat protein is expressed from early double-spliced transcripts and binds to the trans activation response (TAR) region of HIV-1 RNA for its efficient elongation^2^.

Latent infection occurs when the HIV-1 provirus becomes transcriptionally inactive, resulting in a latent reservoir that has become the main obstacle in preventing viral eradication from HIV-1 infected individuals. However, the mechanisms of viral silencing and reactivation remain incompletely understood^3^. Previous studies have suggested that the position of the integration site strongly influences viral gene expression and may be one of the determinants of HIV-1 latency^4^. While highly active anti-retroviral therapy (HARRT) has dramatically decreased mortality from HIV-1 infection, there is currently no effective strategy to target the latent form of HIV-1 proviruses^5^.

Over the last decade, novel genome-editing methods that utilize artificial nucleases such as zinc finger nucleases (ZFNs)^6^ and transcription activator like-effector nucleases (TALENs)^7^ have been developed. These molecularly engineered nucleases recognize and cleave specific nucleotide sequences in target genomes for digestion, resulting in various mutations such as substitutions, deletions and insertions induced by host DNA repair machinery. These technologies have enabled the production of genome-manipulated animals in a wide range of species such as Drosophila^8^, Zebrafish^9^ and Rat^10^. However, ZFNs or TALENs remain somewhat difficult and time-consuming to design, develop, and empirically test in a cellular context^11^. Recently, a third genome-editing method was developed based on clustered regularly interspaced short palindromic repeat (CRISPR) systems. CRISPR systems were originally identified in bacteria and archaea^12^ as part of an adaptive immune system, dependent on a complex consisting of CRISPR RNAs (crRNAs) and CRISPR-associated (Cas) proteins to degrade complimentary sequences of invading viral and plasmid DNA. Mali *et al.* created a novel version of the genome-editing tool applicable to mammalian cells, termed the CRISPR/Cas9 system, which is based on modifications of the *Streptococcus pyogenes* type II CRISPR system in crRNA fused to trans-encoded tracrRNA^13^. This CRISPR/Cas9 system is composed of guide RNA (gRNA) and a human codon-optimized Cas9 nuclease that forms an RNA-protein complex to digest unique target sequences matching those of gRNA. The CRISPR/Cas9 system can be utilized by simple transfection of designed gRNA and a humanized Cas9 (hCas9) expression plasmid into target mammalian cells, making it a promising tool for various applications.

In this study, we tested the ability of the CRISPR/Cas9 system to suppress HIV-1 expression by editing HIV-1 integrated proviral DNA. Cas9 and gRNA, designed to target HIV-1 LTR, were transfected and significantly inhibited LTR-driven expression under the control of Tat. This LTR-targeted CRISPR/Cas9 system can also excise provirus from the cellular genome.

## Results

### LTR-specific editing by CRISPR/Cas9 components disrupts HIV-1 expression machinery

**Figure 1:**
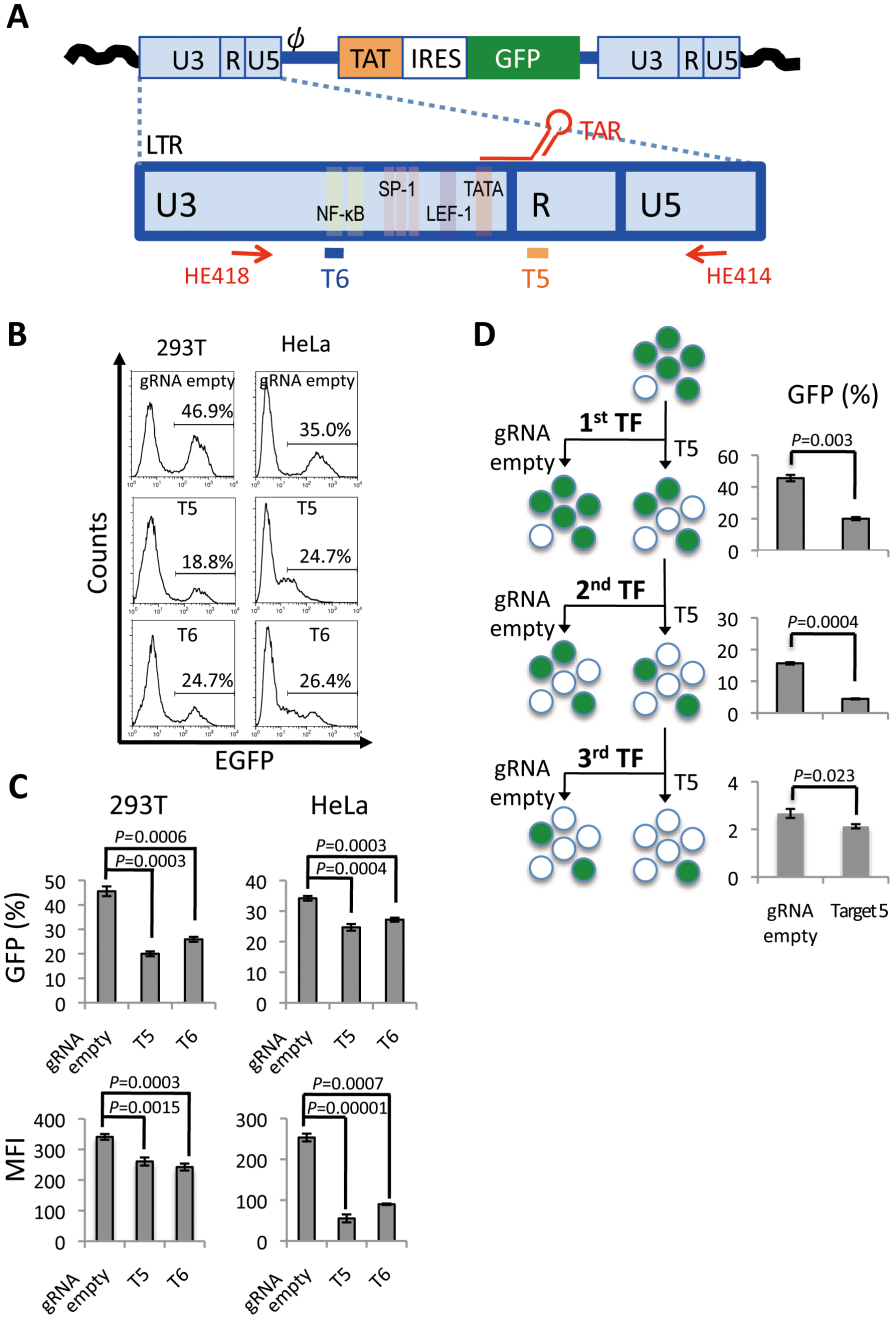
Suppression of HIV-1 gene expression by the CRISPR/Cas9 system targeting HIV-1 LTR. (A) Schematic of provirus derived from LTIG vector and the LTR. Two target sites of CRISPR/Cas9 were indicated at the bottom of the LTR. (B and C) GFP expression after CRISPR/Cas9 treatment. 293 T and HeLa cells were infected with LTIG vector pseudotyped with VSV-G protein. One week after infection, the cells were co-transfected with gRNA expression vector and hCas9 expression vector. The level of GFP expression was analyzed by flow cytometry at 5 days after TF. (B) Representative histograms are shown. (C) GFP positive percentages (top, n = 3), and MFIs (bottom, n = 3) are shown. (D) Percentage of GFP positive 293 T cells after multiple TF with the CRISPR/Cas9 system. Flowchart of the experiment is depicted on the left side. The percentage of GFP positive cells resulting from single, double and triple TF is shown on the right side. The error bars in C and D show standard deviations (n = 3).

We designed a gRNA expression vector to target HIV-1 LTR under the control of the human U6 polymerase III promoter. U6 transcription of gRNA is initiated with guanine and requires the protospacer-adjacent motif (PAM)–NGG followed by a 20-base pair (bp) target sequence^13^. Accordingly, two gRNA-expressing plasmids were generated for targets 5 and 6 (T5 and T6), located in the TAR sequence of the R region and NF-κB binding sequence in the U3 region, respectively (Fig. 1 A), as described in methods. To test the genome editing activity of the CRISPR/Cas9 system, we used HIV-1 provirus-integrated human cells generated by an LTIG HIV vector, which expresses Tat and GFP proteins under the control of an LTR promoter, thus mimicking authentic HIV-1 gene expression^4^. To assess the impact of the CRISPR/Cas9 system targeting HIV-1 LTR, 293 T and HeLa cells were infected with an LTIG vector pseudotyped with VSV-G envelope protein. Then, the LTIG vector-infected cells were co-transfected with a T5 or T6 gRNA expression plasmid together with an hCas9 expression plasmid. Five days after transfection (TF), the mean fluorescence intensity (MFI) of GFP expression and percentage of GFP positive cells were analyzed by flow cytometry. In the 293 T cells, a clear reduction of MFI and GFP positive cells were observed by the CRISPR/Cas9 components (Fig. 1B and C). T5, the TAR-targeting gRNA, was more effective than T6 and reduced the average percentage of GFP positive cells from 45.6% to 20.0% (*p* = 0.0003) (Fig. 1C). Only a modest decline of GFP positive cells was observed in HeLa cells, while the MFI reduction was more drastic than that in 293 T cells (Fig. 1C), probably due to a lower TF efficiency of CRISPR components and a lower level of GFP expression in HeLa cells than in 293 T cells. These results suggested that the HIV-1 LTR targeting CRISPR/Cas9 system blocked HIV-1 gene expression from provirus LTR. Because the most efficient inhibition was obtained by T5 in both 293 T and HeLa cells, T5 was used for the further experiments.

**Figure 2:**
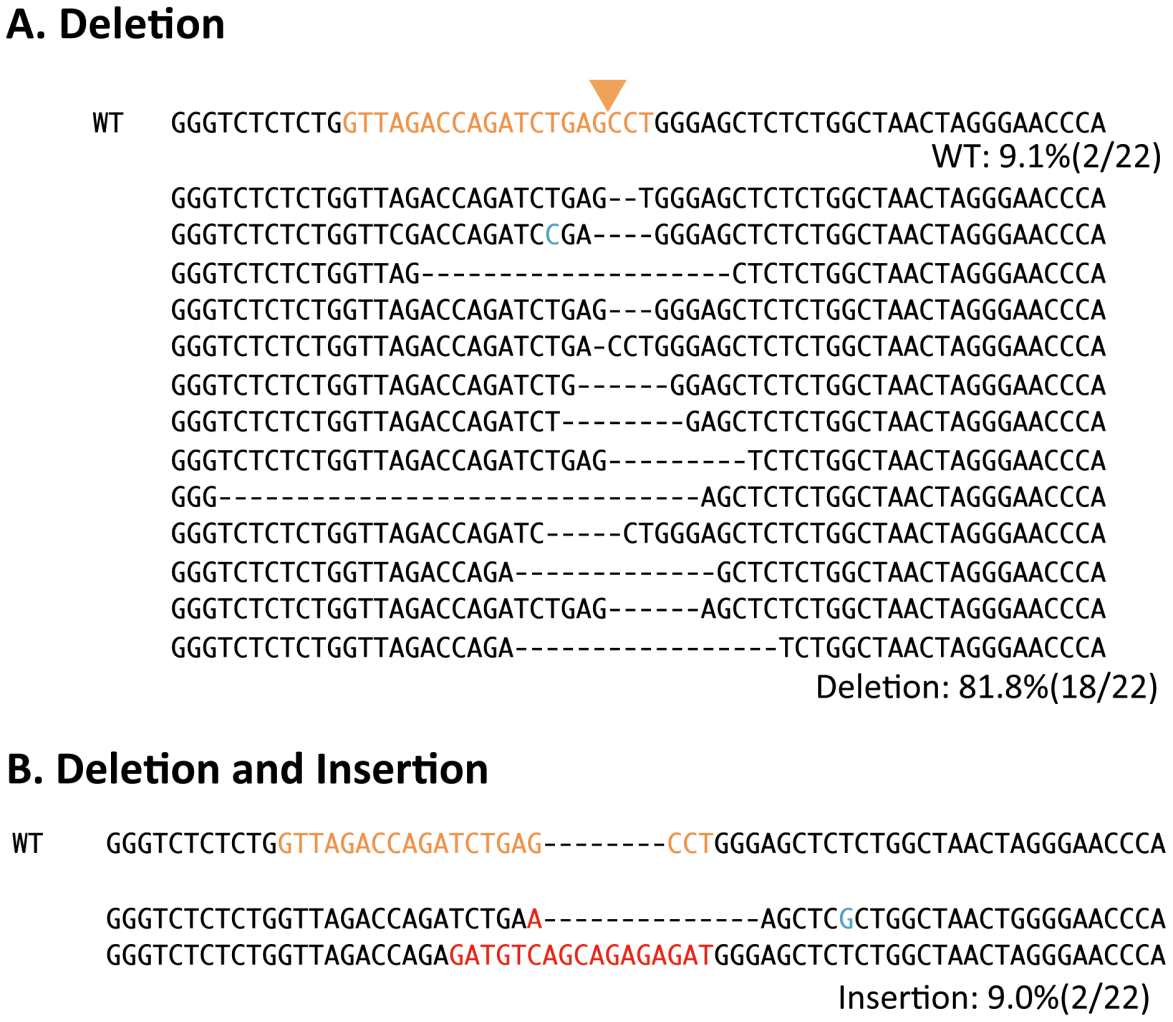
Sequencing analysis of the CRISPR/Cas9-target site. DNA sequence of the TAR region of LTR is indicated. Twenty-two sequences were obtained from LTIG-infected 293 T cells which were transfected three times with T5 gRNA. The WT reference sequence is shown on the top. The target sequence of T5 is indicated in orange. The putative cleavage site is indicated with an arrowhead. (A) TAR sequence with deletions. Eighteen out of 22 clones were deletion mutants. (B) TAR sequence with both deletions and insertions. Two out of 22 clones were mutants consisting of insertion and deletion. Red and blue colors indicate insertion and unanticipated mutation, respectively.

To enhance the inhibition activity of the CRISPR/Cas9 system, we developed a protocol to transfect CRISPR components multiple times. 293 T cells were repeatedly co-transfected with T5 gRNA or gRNA empty and hCas9 plasmids, and flow cytometry analysis was performed five days after TF. As expected, the percentage of GFP positive cells was further reduced after multiple rounds of TF (Fig. 1D). Triple TF resulted in a significant decrease in the mean percentage of GFP positive cells from 40.8% to 2.1% (*p* = 0.0001) was observed. LTR fragments were then isolated from these cells using the primer set as indicated in Fig. 1A and cloned into a plasmid. Sequence analysis of the TAR region of plasmid DNA clones showed that 18 out of 22 HIV DNA clones contained various mutations, between 1 and 31-bp deletions from the end of the putative cleavage site (Fig. 2A). Two clones had a combination of deletion and insertion mutations (Fig. 2B). These mutation patterns are often observed as a result of DNA repair in the non-homologous end joining (NHEJ) pathway and are typical after genome editing^14^, strongly suggesting that this T5 CRISPR/Cas9 component generated double-strand (ds) DNA breaks specifically at the HIV-1 TAR target site, and were repaired through the NHEJ pathway. These results clearly showed that the T5 CRISPR/Cas9 system efficiently produced mutations in the TAR region of proviral DNA.

### CRISPR/Cas9 system can target the latent form of HIV-1 provirus in Jurkat cell

**Figure 3:**
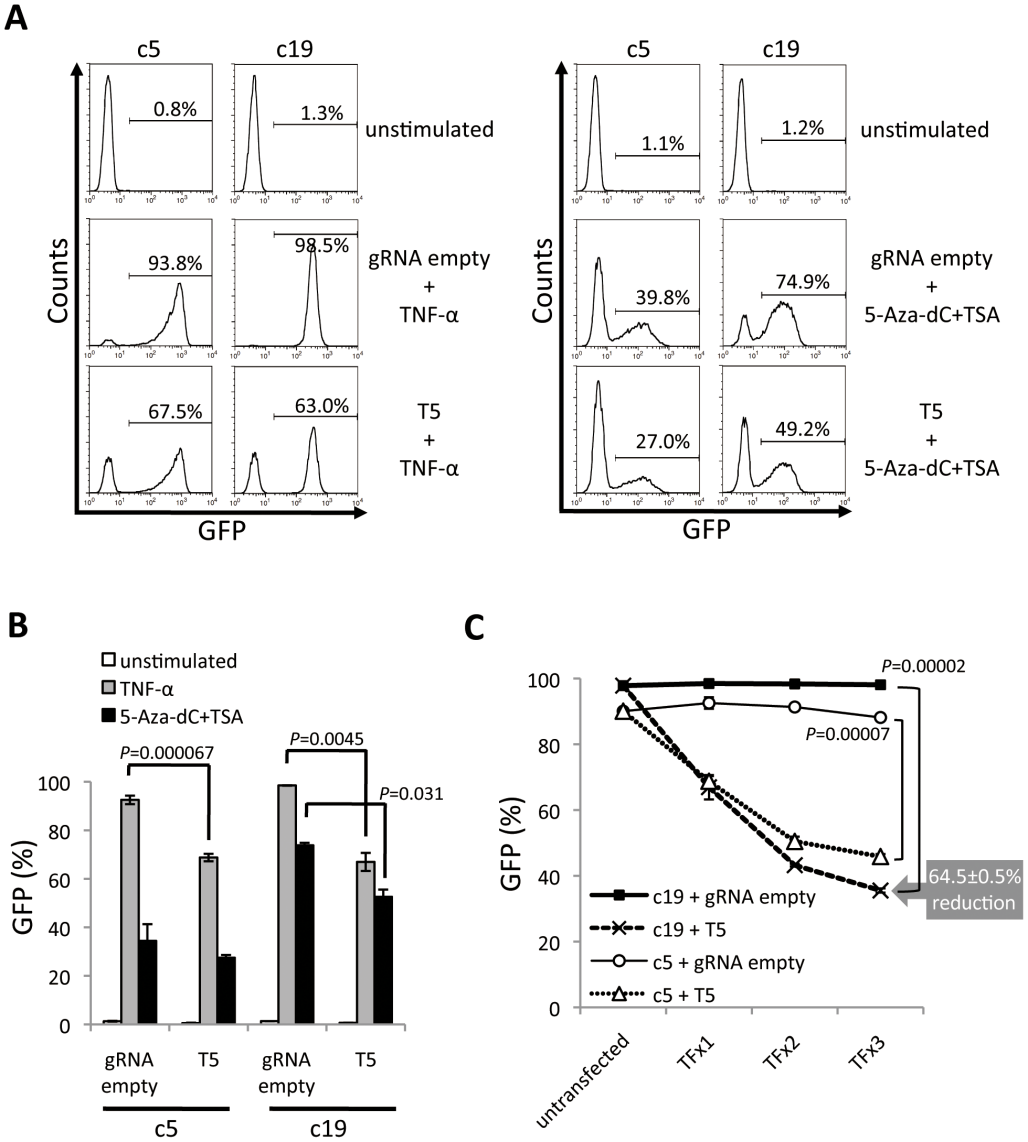
Suppression of proviral re-activation in T cells by the CRISPR/Cas9 system. Jurkat cell lines latently-transduced with LTIG vector were used. These cell lines were co-transfected with gRNA expression vector and hCas9 expression vector. The cells were treated with TNF-α or the combination of 5-Aza-dC and TSA 4 days after TF. (A and B) The level of GFP expression after 48 hours of TNF-α stimulation and 24 hours 5-Aza-dC and TSA stimulation. Representative histograms are shown in A. The positive percentage of GFP is shown in B (n = 3). (C) Multiple TF of T5 or gRNA empty vector and hCas9 expression plasmid. Solid lines with squares and circles indicate c19 and c5 co-transfected with gRNA empty and hCas9 expression vector, respectively. Dotted lines with x and triangles indicate c19 and c5 co-transfected with T5 and hCas9 expression vector, respectively. The error bars in B and C show standard deviations (n = 3).

Because the putative latently infected cells are CD4^+^ T cells, we next tested the genome editing potential of the CRISPR/Cas9 system in these cells. To test this, we generated two Jurkat clone cell lines, c5 and c19, that mimic HIV latency. These cell clones were isolated by limiting-dilution from cell populations that only expressed GFP after induction by either TNF-α or a combination of 5-Aza-dC/TSA. Both c5 and c19 were co-transfected with a T5 or gRNA empty vector and hCas9 expression plasmid. Four days later, cells were treated with TNF-α or a combination of 5-Aza-dC/TSA. The mean percentage of GFP positive cells after TNF-α induction was 92.56% and 98.48% after TF with gRNA empty vector in c5 and c19 cells, respectively. In contrast, the mean percentages of GFP positive cells were 68.78% in c5 and 66.95% in c19 cells, transfected with T5 gRNA (Fig. 3A and B). A similar reduction in GFP expression was observed in c5 and c19 cells transfected with the T5 CRISPR/Cas9 system after 5-Aza-dC/TSA treatment (Fig. 3A and B). These data suggest that the T5 CRISPR/Cas9 system produced cell populations, which were resistant to TNF-α and 5-Aza-dC/TSA stimulation.

To increase the efficiency of the T5 CRISPR/Cas9 system, we performed multiple TF of T5 and hCas9 expression plasmids. This approach significantly reduced the re-activation of latent provirus. As shown in Fig. 3C for c19, the percentage of re-activated latently infected cells was reduced from 97.8% to 35.5% after three rounds of TF (Fig. 3C, *p* = 0.00002). These results clearly demonstrated that the T5 CRISPR/Cas9 system was able to prevent the re-activation of latently integrated provirus in T cells.

### CRISPR/Cas9 system removes HIV-1 internal genes

**Figure 4:**
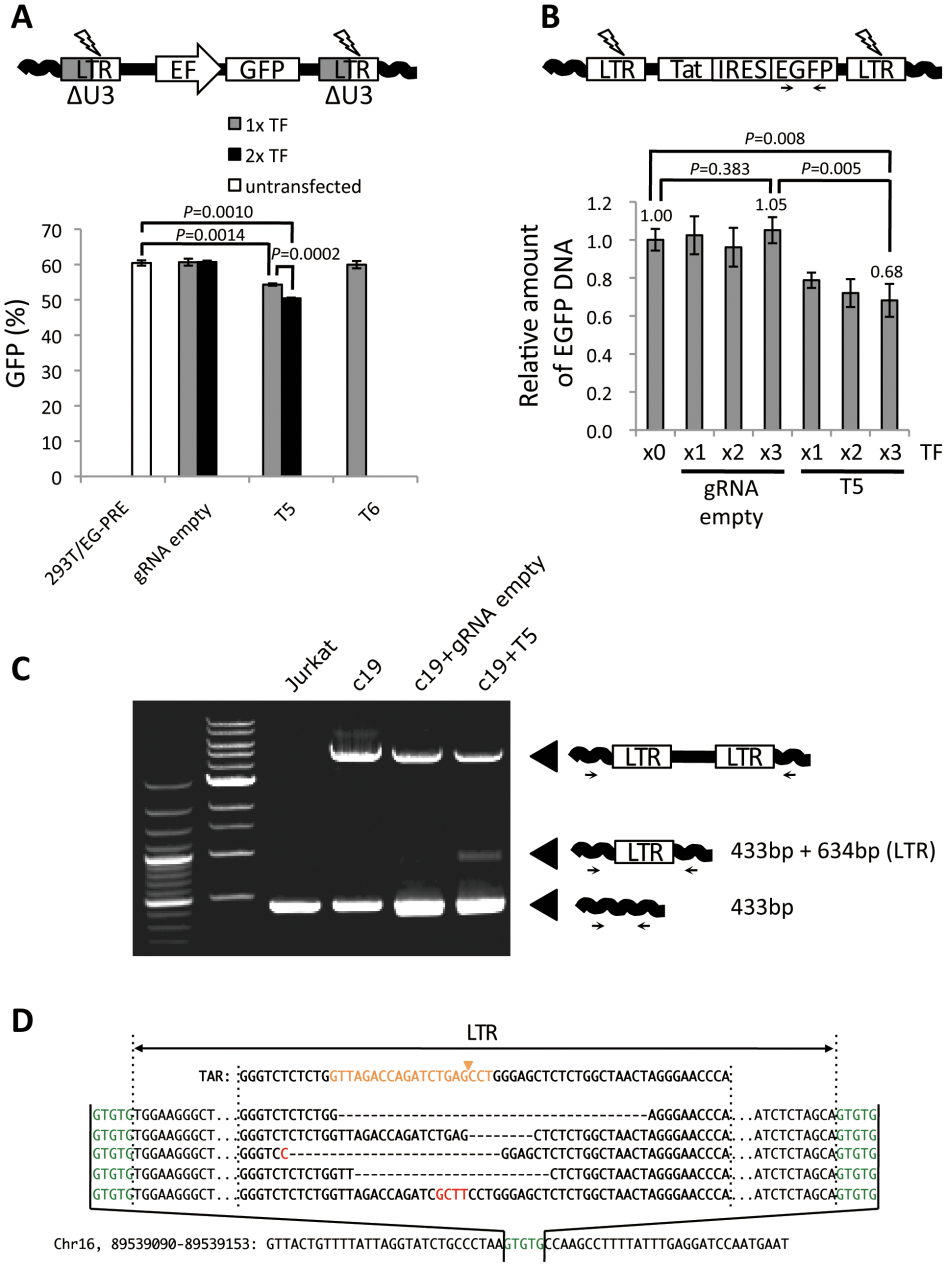
Excision of HIV-1 provirus from host cell genome with CRISPR/Cas9. (A) Excision of lentivirus vector with the CRISPR/Cas9 system. 293 T cells, transduced with lentivirus vector containing an EF promoter derived-EGFP expressing cassette, were treated with the CRISPR/Cas9 system. A schematic of the lentivirus vector used in this assay is shown on top. The percentage of GFP positive cells after single or double TF of CRISPR/Cas9 components is shown on the bottom (n = 3). (B) Excision of LTIG provirus with CRISPR/Cas9 system. The genomic DNA was extracted from the samples derived from Fig. 3C and qPCR was performed (n = 3). The relative amount of *EGFP* DNA is shown. (C) PCR amplification of HIV-1 provirus. A primer set was designed for the host cell genome sequence flanking the proviral integration site in c19. The schematic of PCR products indicating genomic sequences, full-length provirus and one LTR footprint resulting from proviral excision are shown on the right side. (D) DNA sequence of one LTR footprint resulted from proviral excision. TAR sequence of WT LTR is shown on the top in bold. Target sequence of T5 gRNA is indicated in orange. The putative cleavage site is indicated with an arrowhead. LTR sequences from five clones are shown in the middle. Deletions are indicated by dashes. Insertion is shown in Red. The 5-bp direct repeat in host DNA, flanking LTR ends, is shown in green. The host cell genome sequence is indicated on the bottom.

Retrovirus proviral DNA contains duplicate LTR regions on both ends of the integrated viral genome, meaning that the CRISPR/Cas9 system may simultaneously cleave both LTRs and remove an internal region of integrated proviral DNA from the host cell genome. To examine this possibility, we used cells transduced with an alternative HIV-1 vector, missing the U3 region of the LTR and possessing an internal elongation factor-1 (EF) promoter cassette for GFP expression (Fig. 4A). Because GFP expression is driven by an independent EF-promoter, it should be unaffected by the genome-editing system targeting the LTR region of integrated proviral DNA. Therefore, the GFP negative cell populations may be the result of proviral excision. Moreover, since this HIV vector lacks the NF-κB binding site in the U3 region, these cells should be resistant to T6 and not T5-mediated targeting. As expected, only T5 CRISPR/Cas9 components clearly reduced the cell populations expressing GFP (Fig. 4 A, *p* = 0.0014). Double TF of these components resulted in a further decrease of average of GFP positive cells from 60.44% to 50.45% (*p* = 0.0010).

Next, we performed quantitative PCR (qPCR) analysis using c19 Jurkat cells that harbored latently integrated HIV-1 proviral DNA, the LTIG vector. As shown in Fig. 3, the T5 CRISPR/Cas9 system significantly reduced LTR-driven GFP expression. We reasoned that if the inhibition is purely a result of LTR mutations, then, the *EGFP* DNA copy number in the host cell genome should be unchanged following T5 CRISPR/Cas9 treatment. Alternatively, if the reduction of GFP expression is partially attributed to the excision of integrated viral DNA, the amount of *EGFP* DNA will be reduced after T5 CRISPR/Cas9 treatment. For this assay, the c19 cells from Fig. 3C were used. The result of qPCR analysis clearly showed that the relative amount of *EGFP* DNA was decreased by the T5 but not gRNA empty CRISPR/Cas9 treatment (Fig. 4B). On average, 31.8% of the provirus was excised from the host cell genome after CRISPR/Cas9 components were transfected three times. To obtain direct evidence of the provirus-excision effect by CRISPR, PCR analysis using the host cell genome-specific primers, flanking the proviral integration site, was performed. After determining the integration sites of c19, chromosome 16 in latent LTIG-transduced Jurkat cells (Fig. 4D bottom), we designed a primer set specific for both sequences adjacent to the integration site of c19 provirus. The excision of provirus is predicted to leave a footprint of one LTR and the PCR we performed was able to detect the apparent footprint as an additional 1,067 bp fragment only in the T5 CRISPR/Cas9-transfected cells (Fig. 4C). Furthermore, sequence analysis of this PCR product confirmed that excision of provirus resulted in one LTR footprint with a variety of mutations from the cleavage site (Fig. 4D). These results clearly demonstrate that the CRISPR/Cas9 system targeting HIV-1 LTR has the potential to excise latent form of HIV-1 proviral DNA from the host cell chromosome.

## Discussion

In this study, we successfully disrupted the expression of HIV-1 provirus utilizing the CRISPR/Cas9 system (Fig. 1). Importantly, this disruption not only restricted transcriptionally active provirus, it also blocked the expression of latently integrated provirus (Fig. 3). Cas9 proteins are predicted to contain RuvC and HNH motifs^15^, which possess autonomous ssDNA cleavage activity. Interestingly, mutants lacking one of the motifs become nicking endonucleases^16^. It is plausible that the independent nicking activity of each domain may enhance efficient access to the heterochromatin state of latently integrated provirus. Another possibility is that Cas9 has a highly efficient target surveillance system similar to what has been previously reported for the Cas3 system^17^.

T6 gRNA that targeted the NF-κB binding site, also strongly suppressed the LTR promoter activity (Fig. 1). However, the effect was weaker than that of T5 gRNA. In this study we used an LTIG vector modified from the LTR of HIV-1 strain NL4-3 that possesses two adjacent NF-κB binding sites^18^. The T6 target site is at the end of the 5′ NF-κB binding site, meaning that mutations may not completely render transcription inactive since the 3′ NF-κB binding site may remain functional. On the other hand, T5 gRNA that targeted TAR, is profoundly effective in disrupting HIV-1 gene expression. The putative cleavage site was positioned at the neck of the stem loop region of TAR, which is critical for Cyclin T1-Tat-TAR ternary complex formation^19^. Therefore, the TAR sequence may be one of the best targets for blocking HIV-1 provirus expression. Target specificity of the CRISPR/Cas system is very high and a single mutation can disrupt targeting^20^, meaning that some provirus may escape from this genome-editing machinery if mutations arise in target sequences. However, given that the TAR region is relatively conserved and there is little variation among HIV-1 subtypes^21^, it could still be an appropriate target for the elimination of latently infected provirus.

Perhaps the most important finding in this study is that we could excise provirus from the host genome of HIV-1 infected cells, which may provide a ray of hope to eradicate HIV-1 from infected individuals. However, there are numerous hurdles that must be cleared before utilizing genome editing for HIV-1 eradication therapies such as gene therapy. First, the efficiency of genome-editing and/or proviral excision should be quantified in HIV infected primary cells, including latently infected CD4^+^ quiescent T cells. Second, an efficient delivery system must be developed. Fortunately, the CRISPR/Cas9 system has the advantage in size compared with TALENs^22^. Thus, the CRISPR system has the potential to be delivered by lentivirus vectors, whereas TALENs do not because of their large size and repeat sequences^23^. The final hurdle concerns possible off-target effects, which are pertinent concerns for all genome-editing strategies that may lead to nonspecific gene modification events. If Cas9 has off-target effects, then removal of the off-target activity may be the best approach before utilizing CRISPR/Cas system for anti-HIV treatment.

## Methods

### gRNA expression plasmid

gRNA expression plasmids were constructed according to manufacturer's protocol. Briefly, to make a 100 bp dsDNA insert fragment containing the target sequence (20 bp) and PAM sequence, a set of oligonucleotides was used and the fragment was generated by using Phusion polymerase (NEB). The dsDNA fragment was purified and inserted into the AflII site of a gRNA_cloning vector (addgene) with the Gibson assembly system (NEB). Two sets of oligonucleotides targeting 5 and 6 are listed as follows: HE388 (TTTCTTGGCTTTATATATCTTGTGGAAAGGACGAAACACCGttagaccagatctgagcct) and HE389 (GACTAGCCTTATTTTAACTTGCTATTTCTAGCTCTAAAACaggctcagatctggtctaaC); and HE384 (TTTCTTGGCTTTATATATCTTGTGGAAAGGACGAAACACCGctacaagggactttccgct) and HE385 (GACTAGCCTTATTTTAACTTGCTATTTCTAGCTCTAAAACagcggaaagtcccttgtagC), respectively. Lower case letters indicate the target sequence. These 60 nt oligonucleotide sets annealed to each other over a 20 nt complementary sequence at the 3′ ends.

### Virus

Viruses were prepared as described previously^24^. Briefly, 293 T cells were transfected and the culture supernatants were filtrated 48 hours post TF. To prepare VSV-G pseudotyped LTIG vector, pEV731, kindly provided by Dr. Eric Verdin^25^, pMD. G, pMDLg/pRRE and pRSV Rev (helper plasmids) were co-transfected. To prepare VSV-G pseudotyped EG-PRE vector, pCS-CDF-EG-PRE vector and the same helper plasmids were co-transfected as described previously^26^.

### Cell culture

293 T and HeLa cells were maintained in Dulbecco's Modified Eagle Medium (DMEM) containing 10% fetal calf serum (FCS), 100 U/ml penicillin and 100 g/ml streptomycin. Jurkat cells were maintained in RPMI 1640 medium containing 10% FCS, 100 U/ml penicillin and 100 g/ml streptomycin.

### TF and flow cytometry

293 T cells were transfected by the calcium phosphate method^24^. HeLa cells were transfected by Lipofectamine2000 (invitrogen) according to the manufacturer's protocol. Jurkat clone cells were transfected by NEON transfection system (life technology). For TF of CRISPR/Cas9 system, 1 μg of hCas9 expression vector and 1 μg of gRNA expression vector was used. The level of GFP expression was analyzed 5 days after TF. Cells were suspended in phosphate-buffered saline (PBS) containing 1% formamide. Flow cytometry was performed with a FACScCalibur (BD Biosciences), and data were analyzed using CellQuest software (BD Biosciences).

### Establishment of latent form of LTIG-transduced Jurkat cells

Jurkat cells were infected with LTIG pseudotyped vector at MOI 0.5 and cultured for one weak. After treatment with 10 ng/ml TNF-α for 24 hours, GFP positive cells were sorted by FACSAria (BD) and cultured for another one month to relax the GFP expression. Then, GFP negative cells were sorted four times, and the cells were cloned by limiting-dilution. After expanding, clone cells were treated with 10 ng/ml of TNF-α (R&D systems) or a combination of 1 μg/ml 5-Aza-dC (SIGMA-ALDRICH) and 1 μM TSA (SIGMA-ALDRICH), and screened for the potential to be reactivated after stimulation, by flow cytometry.

### Integration-site analysis of latently integrated LTIG provirus

Genomic DNA extracted from Jurkat cell clones were digested with a combination of BamHI and BclI, or NcoI and BspHI and re-ligated by T4 ligase. The ligation products were EtOH precipitated and used as the template for inverse PCR. For this PCR, LA taq (TAKARA) were used according to the manufacturer's protocol. Primer sets used for the inverse PCR were HE410 (CTCCTCGCCCTTGCTCACCA) and M667 (GGCTAACTAGGGAACCCACTGC). The PCR products were cloned into pGEM-T (Promega) vector and sequenced using M13 primers. Primer set, HE433 (AGCACATCACACTCCTCTG) and HE435 (AGACATGAGCCACTATGTCT) were used for PCR amplification of integrated provirus in c19.

### Quantitative analysis of HIV DNA

The amount of *EGFP* DNA was quantified by real-time PCR as previously described^27^,^28^.

### Statistical analysis

All data were expressed as mean ± standard deviations (S.D.). The student's *t* test was used to indicate the differences between groups. *P* values are shown in each figure.

## Author Contributions

H.E. and Y.Koyanagi wrote the main manuscript text. H.E. and N.M. prepared figure 1, 2, 3, 4B, C and D. Y.Kanemura prepared figure 4A. All authors reviewed the manuscript.
